# Supplementary material for: A liquid handling platform for standardized quantification of cell-free enzymatic activity encoded by antimicrobial resistance genes
Source: Synth Biol (Oxf). 2026 Aug 27;11(1):ysag010. doi: 10.1093/synbio/ysag010 (PMC13561876; doi:10.1093/synbio/ysag010)
Supplement: Supplementary file 1 [file args_cfps_sf1_v5_ysag010.docx]

**Supplementary Fig. S1: DMSO concentrations used in cell-free reactions do not statistically impact *E. coli* extract productivity.** Percent inhibition of maximum final DMSO concentrations used in rifampicin assays (0.16% and 0.016%) compared to a water control. Each point represents the median of three technical replicates. One-way t-tests were used to investigate differences between the percentage inhibition of both DMSO treatment groups and the water control. Adjusted p-values (Benjamini & Hochberg correction), shown above each group, indicate no significant differences (α = 0.05).

**Supplementary Fig. S2: Linear relationship between relative fluorescence units (RFU) and mScarlet concentration for five known standards across three independent replicates.** The average linear regression is shown in a black dashed line with a grey ribbon representing standard error.
